# Commissioning of a dedicated commercial Co‐60 total body irradiation unit

**DOI:** 10.1002/acm2.12309

**Published:** 2018-03-11

**Authors:** Jay Burmeister, Adrian Nalichowski, Michael Snyder, Robert Halford, Geoff Baran, Brian Loughery, Ahmad Hammoud, Joe Rakowski, Todd Bossenberger

**Affiliations:** ^1^ Karmanos Cancer Center Gershenson R.O.C. Detroit MI USA; ^2^ Department of Oncology Wayne State University School of Medicine Detroit MI USA; ^3^ William Beaumont Hospital – Dearborn Dearborn MI USA

**Keywords:** total body irradiation, TBI, Co‐60 teletherapy

## Abstract

We describe the commissioning of the first dedicated commercial total body irradiation (TBI) unit in clinical operation. The Best Theratronics GammaBeam 500 is a Co‐60 teletherapy unit with extended field size and imaging capabilities. Radiation safety, mechanical and imaging systems, and radiation output are characterized. Beam data collection, calibration, and external dosimetric validation are described. All radiation safety and mechanical tests satisfied relevant requirements and measured dose distributions meet recommendations of American Association of Physicists in Medicine (AAPM) Report #17. At a typical treatment distance, the dose rate in free space per unit source activity using the thick flattening filter is 1.53 × 10^−3^ cGy*min^−1^*Ci^−1^. With a 14,000 Ci source, the resulting dose rate at the midplane of a typical patient is approximately 17 and 30 cGy/min using the thick and thin flattening filters, respectively, using the maximum source to couch distance. The maximum useful field size, defined by the 90% isodose line, at this location is 225 × 78 cm with field flatness within 5% over the central 178 × 73 cm. Measured output agreed with external validation within 0.5%. End‐to‐end testing was performed in a modified Rando phantom. In‐house MATLAB software was developed to calculate patient‐specific dose distributions using DOSXYZnrc, and fabricate custom 3D‐printed forms for creating patient‐specific lung blocks. End‐to‐end OSLD and diode measurements both with and without lung blocks agreed with Monte Carlo calculated doses to within 5% and in‐phantom film measurements validated dose distribution uniformity. Custom lung block transmission measurements agree well with design criteria and provide clinically favorable dose distributions within the lungs. Block placement is easily facilitated using the flat panel imaging system with an exposure time of 0.01 min. In conclusion, a novel dedicated TBI unit has been commissioned and clinically implemented. Its mechanical, dosimetric, and imaging capabilities are suitable to provide state‐of‐the‐art TBI for patients as large as 220 cm in height and 78 cm in width.

## 1 INTRODUCTION

Total body irradiation (TBI) is commonly used as a preparatory regimen for bone marrow transplant for disseminated cancer. American Association of Physicists in Medicine (AAPM) Report #17 “Aspects of Total Body Irradiation” describes the requirements and recommendations for TBI.^1^ Since most radiotherapy centers perform relatively few such treatments, typical TBI involves adaptation of conventional radiotherapy equipment and treatment procedures to facilitate irradiation of the whole body. A common example is the use of a conventional linac rotated to deliver a lateral beam irradiating a patient placed a large distance away from the isocenter to create a field large enough to accommodate the entire patient. In many cases, the field size is still not large enough and the patient must be treated in the fetal position. Aside from the complexity of the treatment setup, the use of lateral fields is not optimal since the patient is thicker laterally than anteroposteriorly, resulting in greater dose heterogeneity. In addition, shielding the lungs to mitigate radiation pneumonitis, the most important common toxicity resulting from TBI, is much easier using anterior/posterior fields. Due to the difficulties accommodating TBI treatments using conventional radiotherapy treatment units, and the associated suboptimal treatment characteristics, some facilities performing large numbers of TBI treatments have devised dedicated TBI units by modifying existing clinical equipment to overcome these shortcomings. Some examples of these units are described in the literature using either linear accelerators^2^, ^3^, ^4^, ^5^ or Co‐60 units.^6^, ^7^, ^8^^.^

In 1994, our center modified a commercial Co‐60 teletherapy unit (Theratron 780, Atomic Energy of Canada Ltd., Chalk River, Canada) to create a dedicated TBI unit similar to that described by Peters, et al.^9^ Modifications included removal of the collimator and treatment couch and the construction of a custom flattening filter. A thin, movable couch with adjustable lung block tray was designed to support the patient a few cm off the floor with a gap underneath large enough to allow the assessment of lung block positioning with radiographic film. The surface of the couch was approximately 190 cm from the source. The removal of the collimator allowed the entire patient length to be included in the treatment fields. Patients were treated with an AP and a PA field using a supine and prone setup, respectively, and the custom flattening filter was designed to produce a uniform dose distribution over the lateral and longitudinal axes of the patient. This unit was well suited to provide uniform total body dose distributions and facilitate appropriate lung blocking and was operated from 1994 to 2016, delivering TBI treatments to over 700 patients in preparation for bone marrow transplant. In 2016, the GammaBeam 500 (Best Theratronics, Inc., Kanata, ON, Canada) became the first FDA approved commercial dedicated TBI unit. Its design is very similar to our previous in‐house unit, however, it has greatly improved user interfaces, the height of the treatment head is adjustable, it projects a significantly larger field size, and the treatment couch includes an amorphous silicon flat‐panel imaging device. Our in‐house developed dedicated Co‐60 TBI unit was de‐commissioned and replaced with this unit in October 2016. This work describes the commissioning processes and results for the first commercial dedicated TBI unit in clinical operation.

## 2 MATERIALS AND METHODS

**Figure 1:**
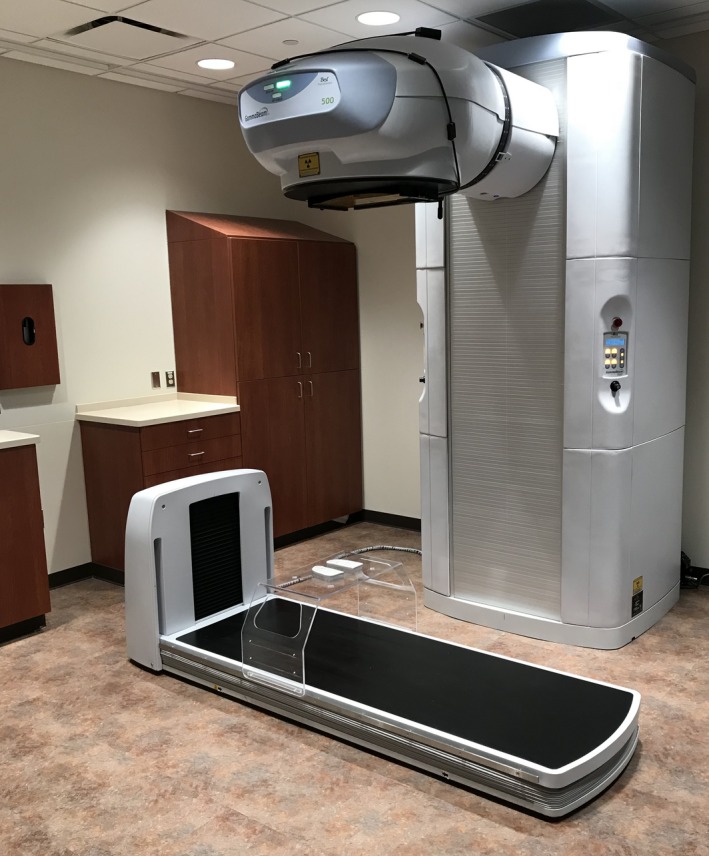
Photograph of the GB500 treatment unit and couch along with lung blocks placed on block tray.

The GammaBeam 500 Total Body Irradiator is a Co‐60 teletherapy unit designed to deliver a large field size at extended distance for TBI. The treatment beam points toward the floor and the head moves vertically with a minimum and maximum distance of 74 and 250 cm from the source to the floor, respectively. As such, both the field size and dose rate are variable. Patients are treated with AP and PA fields on a movable treatment couch with built‐in imaging capabilities. While the unit allows rotation of the treatment head, we have disabled beam operation if the head is not locked such that the beam points vertically downward. A blocking tray which attaches to the couch can hold custom lung blocks at three different heights above the treatment couch. The couch has motorized vertical motion to allow easy patient access prior to lowering the couch to the treatment position, and a 41 × 41 cm a‐Si flat panel imager with motorized longitudinal motion which can be used for positional verification of the patient and/or blocks. The couch is interlocked such that treatment can only be performed when the couch is at its lowest position. The manufacturer specified treatment field at a distance of 220 cm from the source is 70 × 200 cm. A photograph of the unit is shown in Fig. 1.

While comprehensive recommendations for commissioning and clinical implementation are regularly issued for conventional radiotherapy treatment units and techniques, this is not the case for unique or specialized treatment units such as that described here. Indeed, AAPM Report #17 on TBI was published in 1986 and no updated AAPM recommendations have since been issued. As a result, limited guidance exists for the commissioning, clinical implementation, and quality assurance of treatment units such as this. Our first goal in the commissioning process was to assure the safe use of the treatment unit and confirm the proper operation of all treatment unit functions. This included a radiation survey; testing of all safety interlocks, emergency systems, and radiation indicators; development of quality assurance tests and frequencies; development of staff training documentation; and a failure mode and effects analysis.

Following the development and testing of all safety processes, we evaluated the operation and accuracy of all mechanical systems within the treatment unit, couch, and imaging system. Accuracy and reproducibility of the treatment head rotation and locking mechanism and flattening filter position and reproducibility were evaluated along with their resulting effects on the characteristics of the treatment field. Source translation accuracy and reproducibility testing included repeated calibration measurements for a fixed treatment time, measurement of timer linearity and magnitude and reproducibility of timer error, and reproducibility of measured time for each phase of source translation. These phases represent the time to trigger successive microswitches in the source translation processes and are referred to as the “fully shielded”, “just shielded”, and “fully exposed” phases of both the “exposure” and “return” processes. The GammaBeam 500 treatment console provides access to the source translation time for each phase of translation with an accuracy of 10 ms. This allows a more sensitive evaluation of the reproducibility of the source translation process than simple measurement of the timer error. While the vertical position of the treatment head is adjustable, we decided for simplicity and safety to treat all patients with a single head height. In order to achieve the largest possible field size, we chose that height to be the maximum head height which is 247.0 cm from the source to the floor and 228.6 cm from the source to the surface of the treatment couch. This results in a typical source to surface distance (SSD) of approximately 207 cm for patient treatment. Treatment couch operation, couch sag, and couch motion were evaluated with loads of 250 and 400 pounds.

**Figure 2:**
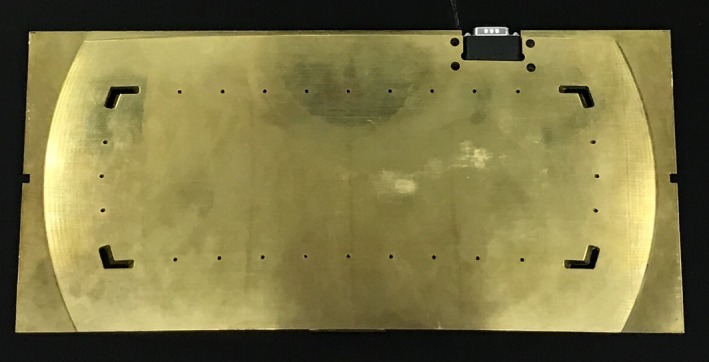
Photograph of the attenuating flattening filter showing the filter shape and the light holes for field definition.

Radiation output characteristics were evaluated, including coincidence of light and radiation fields, measurement of absolute dose rate in free space and in phantom, output as a function of distance from the source and as a function of position in the plane perpendicular to the beam axis, tissue‐air ratio (TAR) measurements, external dosimetric validation, and the development of in‐vivo dosimetry processes. The light field for this unit is projected using a series of divergent holes in the periphery of the flattening filter. These holes are visible in the photograph in Fig. 2. We evaluated light/radiation coincidence on the treatment room floor as well as on the couch at the patient treatment level. In addition, we evaluated the effects of the light field holes on the dose distribution.

Absorbed dose rate in free space was measured using a Farmer chamber (PTW, Freiburg, Germany) with a 5 mm buildup cap at a distance of 30.5 cm above the couch (198.1 cm from the source) in order to avoid the effects of backscatter from the couch and floor. This dose rate measurement was repeated using a separate Farmer chamber as well as a small volume (0.125 cm^2^) scanning chamber (PTW, Freiburg, Germany). These chambers were also used to evaluate the output as a function of distance from the source over the range of 173.2–218.6 cm source to chamber distance. Off‐axis output measurements were made as a function of position from the beam central axis both in‐air using the Farmer chamber with buildup cap, and in‐phantom using the Farmer chamber at the center of a 20 cm deep stack of solid water with a surface area of 40 × 40 cm. Measurements were made both with and without the treatment couch present and effects of scatter from the couch and from the head of the couch (the housing for the couch vertical drive mechanism) were evaluated. We measured the uniformity of the radiation field along the longitudinal axis up to 120 cm from the central axis in both directions and along the transverse direction up to 50 cm from the central axis in both directions. We also measured a diagonal profile through the corner light field holes as well as an array of point measurements within each quadrant of the treatment field.

Absolute calibration was performed using a Farmer chamber within a 60 × 60 cm^2^ water phantom filled to a depth of 30 cm with 40 × 40 × 30 cm^3^ solid water placed on either side of the water phantom in the longitudinal direction. Since the field size is not adjustable we cannot create a 10 × 10 cm field size, and since we don't have a phantom large enough to cover the single fixed field size, we are not able to achieve full scatter. As such, we are not able to perform calibration per AAPM TG‐51 guidelines,^10^ however, the formalism of the TG‐51 report was followed as closely as possible. In addition, we followed the recommendations from AAPM Report #17 section 3.1 which state that one should (1) perform absolute calibration using large field geometry and the largest phantom possible, (2) correct for (a) dose that would be obtained for a phantom that covers the entire beam, and (b) dose that would be obtained within a deep phantom (full scatter), and (3) correct for patient dimensions in terms of both area and thickness when performing calculations for patient treatment. The unit has only one field size and this phantom is the largest we could create with existing equipment. We performed the recommended corrections to our measurements using Tables 2 and 3 from AAPM Report #17. Since Table 2 is limited to a field size of 75 × 75 cm^2^ and a phantom size of 50 × 50 cm^2^ it was necessary to extrapolate to accommodate our setup. Our equivalent field size as defined by the 50% isodose line in air of 250 × 85 cm is approximately 125 × 125 cm^2^. Our equivalent phantom size for the 120 × 30 × ~55 cm is approximately 75 × 75 cm^2^.

Tissue‐air ratio data for our TBI program have traditionally been based on percent depth dose (PDD) data from british journal of radiology Supplement 11^11^ modified to include larger field sizes and to better match in‐phantom measurements. These TAR values match very well (within 0.3% for typical field sizes) with values from Table 1 from van Dyk et al.^12^ Since our largest phantom is not sufficient to provide infinite scatter for this field size, we are unable to directly measure TARs or PDDs for this treatment unit. Instead, we performed a set of representative measurements to determine whether the previously used TAR values are accurate for this treatment unit. This was performed for phantom geometries smaller than the total field size and similar to those of typical patients. PDDs measured in this phantom were converted to TARs using the formula: TAR(d,A’)=(PDD(d,A,f)/100)xPSF(A)x[(f+d)/(f+dmax)]^2^. Recommendation (3) from AAPM Report #17 section 3.1 represents the correction from full scatter dose calculations to the finite patient size and these correction values are interpolated and applied past 10 cm depth. Measurements were made at depths of 0.2–20 cm for phantom equivalent square field sizes of 50 × 50 cm^2^, 60 × 60 cm^2^, and 75 × 75 cm^2^.

**Table 1:** Calculated and measured doses on the end‐to‐end phantom at a depth of 1 cm for an AP field delivering 100 cGy

Location	Calculated dose		Measured dose	
	Manual	Monte Carlo	Diode	Diode/MC
Head	125.8	134.3	133.3	0.99
Suprasternal notch	132.6	137.5	135.3	0.98
Abdomen	141.3	145.0	140.8	0.97
Knees	132.3	135.4	136.0	1.00
Ankles	119.0	127.2	126.8	1.00

External dosimetric validation was performed through the Imaging and Radiation Oncology Core (IROC) at the University of Texas MD Anderson Cancer Center. Optically Stimulated Luminescence Detectors (OSLDs) were irradiated using the standard IROC acrylic irradiation block and setup. In addition, we made measurements using an identical acrylic calibration block with a hole drilled for a 0.053 cm^3^ ionization chamber (Exradin A1SL, Standard Imaging, Middleton, WI, USA). The dose to muscle tissue in the calibration setup was calculated from the ionization chamber measurement and compared to the dose to muscle tissue calculated from the OSLD measurement.

In‐house processes were developed for treatment planning and for the creation of lung blocks^13^ which are required to reduce the likelihood of radiation pneumonitis. Full‐length CT scans of the patient are created by joining separate scans of the superior and inferior halves of the patient. This is performed for both supine and prone setups to allow calculation of both AP and PA full body treatment fields. These scans are imported into the Eclipse v11.0 treatment planning system (Varian Medical Systems, Palo Alto, CA, USA) for field definition, and lung block shapes are drawn if blocking is necessary. The planning geometry and image data are then sent to an in‐house developed MATLAB (Mathworks, Natick, MA, USA) program designed to initiate a DOSXYZnrc 14 simulation based on patient specific anatomy and lung blocking (if necessary).^13^ The calculated dose distribution can then be exported back to Eclipse for plan evaluation. Information describing lung block shapes drawn in Eclipse is extracted from the DICOM plan information and used to create mold models for the lung blocks which are 3D printed using a polylactic acid (PLA) thermoplastic. The PLA molds are then filled with Lipowitz's alloy to create the lung blocks and the molds subsequently removed and discarded.^13^

End‐to‐end testing was performed for the treatment simulation, planning, lung block creation, and treatment delivery processes using a Rando phantom (Alderson Research Laboratories, Stamford, CT, USA) modified by adding arms and legs made from acrylic tubes filled with water. Gafchromic EBT3 + film (Ashland, Wayne, NJ, USA) was placed axially between phantom slices in the abdomen, thorax, and head. NanoDot OSLDs (Landauer, Glenwood, IL, USA) were placed inside the phantom adjacent to each film, one in the center of the head, one in the center of the abdomen, one in the center of the right lung, and one at the lung/chestwall interface in the right lung. All OSLDs were read in‐house using a Microstar ii OSLD reader (Landauer, Glenwood, IL, USA). Diode measurements were performed on the phantom surface on the head, suprasternal notch, abdomen, knees, and ankles using an Isorad (CNMC, Inc., Nashville, TN, USA) diode with inherent 1 g/cm^2^ buildup. The abdomen location was 20 cm superior to the longitudinal center of the patient and serves as the approximate location of maximum equivalent field size. The knee and ankle measurement locations were estimated based on typical patient measurements.

The GammaBeam 500 treatment couch provides a 240 × 78.5 cm treatment surface with a movable 41 × 41 cm a‐Si imaging panel underneath the carbon fiber couch surface. The mechanical and imaging capabilities of the imaging subsystem were tested for patient setup and for positioning of lung blocks. The imaging system was calibrated for various combinations of gain (0.5–8 pF), integration time (133–1000 ms), and exposure time (0.01–0.1 min). These combinations were used to quantitatively evaluate image quality using a contrast‐detail phantom,^15^ and then used to image the Rando phantom with and without lung blocks to qualitatively evaluate the accuracy of lung block placement.

Finally, we developed a set of routine quality assurance tests associated with the dosimetric, mechanical, and radiation safety aspects of the unit and its operation. These tests, along with the associated tolerance and frequency, were based on regulatory requirements of the United States Nuclear Regulatory Commission (USNRC), AAPM recommendations for other radiotherapy delivery devices, and standard departmental procedures.

## 3 RESULTS

All radiation survey results were within requirements both inside and outside the treatment room. The exposure rate was measured at 18 positions around the treatment unit head shielding with a maximum exposure of 18 mR/h at the surface of the shield and maximum and mean of 4.0 and 0.9 mR/h at 1 m from the source position. The treatment head contains an emergency shutter which closes over the treatment field in the case that the source rod is stuck in the exposed position. While there is no mechanism to intentionally expose the source with the shutter closed to measure shutter transmission, leakage measurements through the shutter in the factory in air at 220 cm from the source resulted in an exposure rate of 3.6% of the open field. All safety and emergency features, interlocks, and indicators functioned as designed.

The most significant potential deviation between intended and delivered dose for treatment delivery using this unit is likely the patient position with respect to the source. While the treatment couch is movable in the vertical direction over a range of 46 cm, the treatment beam is interlocked such that the source will immediately retract if, at any point, the couch is not in its lowest position. Since the head height is variable over approximately 175 cm, a head height (“Z‐axis”) interlock is incorporated to assure that the source to patient distance is set as intended. In addition, laser‐based SSD measurement incorporated in the treatment unit is used prior to each treatment field to assure expected SSD and thus proper patient setup. The tolerance of both the head height and couch vertical position interlocks was measured to be less than 2 mm. This results in a potential error of less than 0.2% given the typical treatment SSD of greater than 200 cm.

When the head is locked at the 0° rotation position, both the faceplate and flattening filter surface show a rotation of between 0.3° and 0.4° counterclockwise from horizontal facing the gantry and this is consistent within 0.1° over repeated unlocking, rotation, and relocking. Repeated rotation and relocking resulted in an overall variability of approximately 8 mm in the measured longitudinal position of the light field projected on the floor. This longitudinal variability corresponds to approximately 0.2° of variability in head rotation given this light source to floor distance. This affects the overall accuracy of off‐axis beam characteristics in the patient S‐I direction. A similar test of reproducibility of the flattening filter location resulted in approximately 1 mm variation in the light field projected on the floor and affects the overall accuracy of off‐axis beam characteristics in the patient R‐L direction. These uncertainties are included when assessing the maximum patient width and length for uniform treatment. At the lowest couch position, couch sag was negligible with a patient load of 250 lbs and approximately 2 mm with a patient load of 400 lbs.

All source translation times were evaluated at the time of commissioning and each month thereafter. Over the first 6 months of operation, all measured source translation times for each translation phase have remained within 20 ms and/or 3% of the mean value. The timer error was measured using the method of Orton 16 to be 0.013 min during all timer error measurements performed from the time of commissioning to the present. Timer accuracy was measured to be better than 0.01 min and timer linearity was measured to be within 0.1% from 30 s to 15 min.

The unit offers both a thick (named “attenuating”) and a thin (named “non‐attenuating”) flattening filter, allowing the user a choice of two dose rates. For the maximum treatment head height of 247 cm, the dose rate in free space measured using the attenuating flattening filter at the time of initial calibration at a distance of 198.1 cm from the source was 23.3 cGy/min for a source activity of 13,838 Ci, or 1.69 × 10^−3^ cGy min^−1^ Ci^−1^. At a distance representative of the midplane of a typical patient, or approximately 218 cm from the source, the dose rate in free space at the time of initial calibration was 1.53 × 10^−3^ cGy min^−1^ Ci^−1^. The corresponding dose rate inside a typical patient at midplane is approximately 17 cGy/min using the attenuating flattening filter and 30 cGy/min using the non‐attenuating flattening filter. As such, the total exposure time for a 2‐Gy fraction delivered through AP and PA fields is approximately 12 min with the attenuating filter. In an attempt to most closely match our previous clinical experience and thus avoid any potential increase in pneumonitis or graft versus host disease due to a significant increase in dose rate, 17, 18 we chose to commission the treatment unit using the attenuating filter. When the source activity has decreased to a level requiring treatment times that are uncomfortable for patients, we will recommission the treatment field using the non‐attenuating filter. *In vivo* dosimetry is performed for each treatment field by performing diode measurements on the patient surface for the first minute of the treatment field, thus leaving time to adjust the treatment time in the case that the measured dose deviates from the expected dose. The diode is calibrated in the Co‐60 beam under conditions similar to patient irradiation and our clinical tolerance for the *in vivo* measurements is ±8%.

Independent validation measurements of the dose rate in free space were performed using a separate Farmer chamber and 0.125 cm^3^ scanning chamber and were within 0.2% and 0.4% of the initial measurement, respectively. In‐air measurements performed as a function of distance from the source followed an inverse square relationship within 1% to within 10 cm of the couch top, and in phantom measurements at d_max_ with 10 cm backscatter followed an inverse square relationship within 0.3% to the couch top.

**Figure 3:**
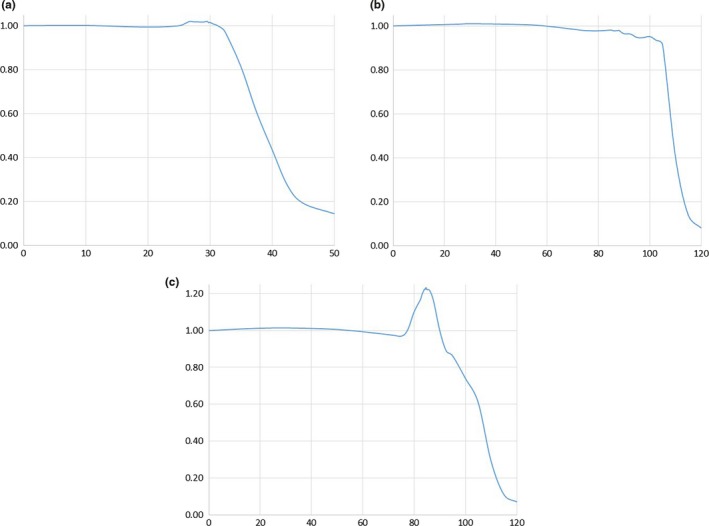
Beam profiles measured in free space in a plane perpendicular to the beam central axis 178.6 cm from the source along the (a) transverse (b) longitudinal, and (c) diagonal directions.

Figure 2 shows an image of the attenuating flattening filter. The holes in the filter allow projection of the light field and define the approximate usable field size. Figures 3(a)–3(c) show the characteristics of the transverse, longitudinal, and diagonal beam profiles measured in free space at a distance of 178.6 cm, or 50 cm above the couch. Only half of each profile is shown to allow better visualization of the field characteristics. All symmetric points within all three profiles are within 2%.

While the light field holes allow visualization of the field size, they should ideally not significantly affect the characteristics of the treatment beam. At the distance of these measured profiles, the light field holes project to approximately 30 cm and 88 cm in the transverse and longitudinal axes, respectively. A maximum increase of approximately 2% above the central axis output is observed in the transverse profile, while the longitudinal profile does not show an increase over the central axis output since the light hole is near the penumbral region. In comparison, an increase of nearly 25% is observed in Fig. 3(c) underneath the corner light holes.

The usable size of the treatment field based on measurements in the center of a 20 cm thick solid water phantom placed on the couch is 225 cm in the longitudinal direction and 78 cm in the lateral direction. More specifically, this field size is defined by the 90% isodose contour measured at 10 cm depth in phantom with 20 cm of scatter on all sides of the phantom in a plane perpendicular to the beam axis and 218.6 cm from the source along the beam central axis. The field flatness is within 5% over the central 178 × 73 cm for these measurement conditions. These treatment beam field sizes can be compared to the field defined by the light field holes at 218.6 cm from the source which is approximately 214 cm in the longitudinal direction and 74 cm in the lateral direction.

**Figure 4:**
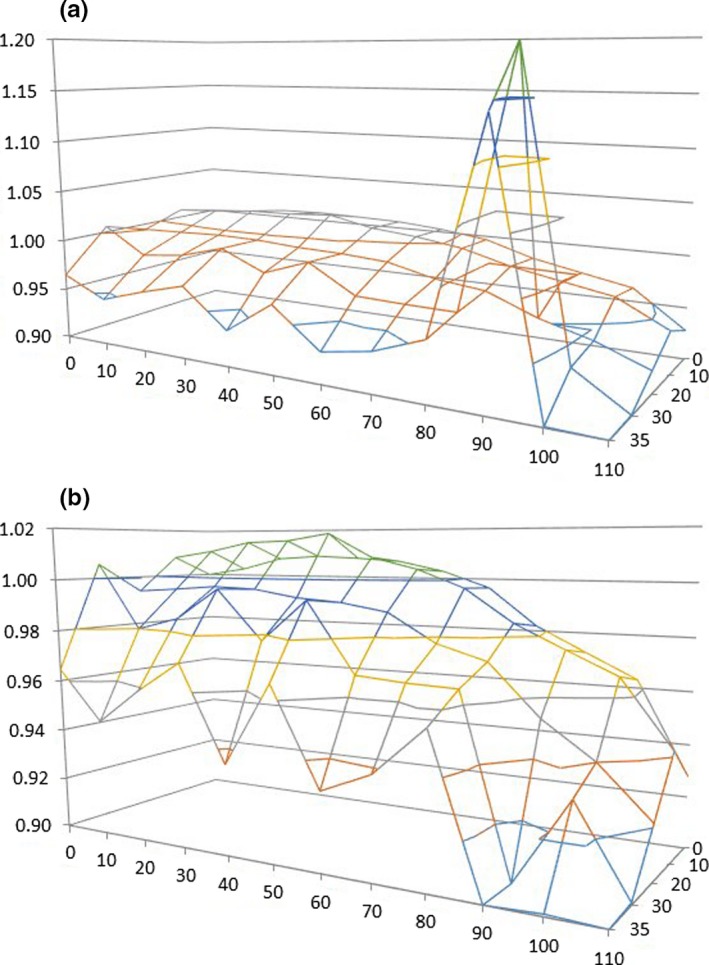
Beam profiles measured in free space in a plane perpendicular to the beam central axis 198.1 cm from the source (a) with and (b) without measurement points under corner light field hole.

In addition to measurements made along the primary beam axes, we made measurements to confirm the flatness of the remainder of the treatment field. Since the source, primary collimation, and flattening filter are all symmetric, we performed full point dose measurements in one quadrant only and spot checked the other three quadrants. Figures 4(a) and 4(b) illustrate the beam characteristics in this region. With the exception of the points under the corner light hole, all measured doses in this quadrant are within 5% of the central axis dose to 90 cm in the longitudinal direction and 30 cm in the lateral direction, and within 10% to greater than 100 cm in the longitudinal direction and 35 cm in the lateral direction. Figure 4(b) shows the beam characteristics with the corner light hole positions eliminated to better illustrate the shape of the profile in two dimensions. All point measurements in the other three quadrants were within 1.5% of those in the fully measured quadrant shown here.

All TAR values calculated from measurements made at depths of 0.2–20 cm within the 50 × 50 cm^2^, 60 × 60 cm^2^, and 75 × 75 cm^2^ equivalent square phantoms were within 2.0% of those used for our previous in‐house modified treatment unit and from Table 1 of van Dyk et al.^12^ These values are further validated within the end‐to‐end testing results, as the manual calculations presented here use the dose rate in free space multiplied by the appropriate off‐axis factors and the TAR for the estimated equivalent square field size of the phantom at each anatomic location. Our reported dose at d_max_ for the external validation agreed to within 0.1% of the IROC measured dose at d_max_.

End‐to‐end testing was performed initially without lung blocking. Table 1 shows calculated and measured doses along the surface of the phantom for a prescribed delivered dose of 100 cGy from the AP treatment field only. Table 2(a) shows calculated and measured doses at various locations at depth within the phantom for a prescribed total delivered dose of 200 cGy from both AP and PA fields. All measured absorbed doses without lung blocking agree with calculations to within 4%. Measured mean absorbed dose and standard deviation for the abdomen and head film measurements were 194.3 ± 7.9, and 199.4 ± 8.7 cGy, respectively. These results illustrate the accuracy and relative uniformity of the dose distribution within these slices, with approximately 96% and 98% of pixels within ±10% of the prescribed dose in the abdomen and head films, respectively.

**Table 2:** Calculated and measured doses at various locations within the end‐to‐end phantom for a prescribed total delivered dose of 200 cGy from AP/PA fields (a) without and (b) with 2.5 cm thick lung blocks

Location	Calculated dose		Measured dose	
	Manual	Monte Carlo	OSLD	OSLD/MC
(a) Without 2.5 cm thick lung blocks				
Head	201.2	197.5	204.9	1.04
Chest wall	220.4	220.2	218.6	0.99
Head	231.7	230.2	228.6	0.99
Abdomen	199.8	194.4	197.4	1.02
(b) With 2.5 cm thick lung blocks				
Head	201.2	197.7	206.6	1.05
Lung	86.9	83.8	84.0	1.00
Abdomen	199.8	196.2	197.2	1.01

**Figure 5:**
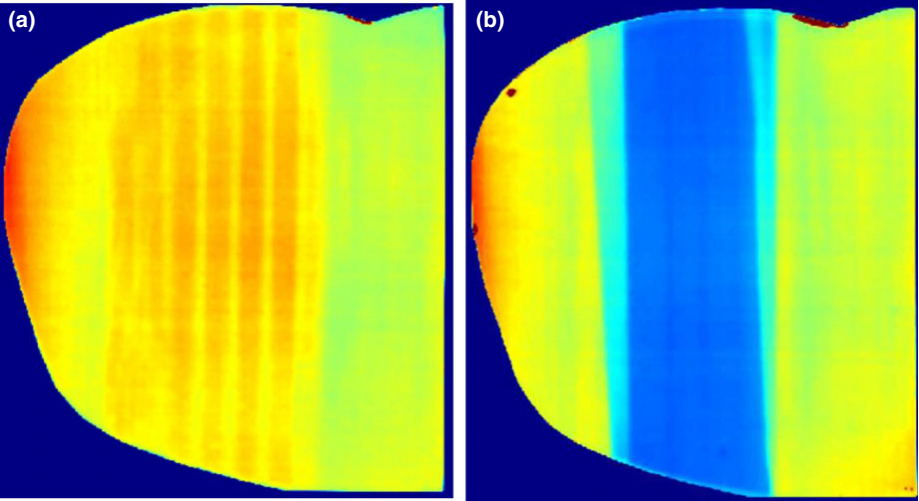
Axial film dose distributions within the thorax (a) without and (b) with lung blocking.

Figures 5(a) and 5(b) show the thorax films both with and without lung blocking. The thorax film shows both a higher mean dose due to the decreased attenuation and a larger standard deviation resulting from the non‐uniformity of the phantom material and the unit density plugs placed throughout the lung material which can be removed to allow placement of dosimeters. The decreased attenuation within the lung and higher dose streaks from the more attenuating plugs are clearly observed in the film acquired without lung blocking.

**Figure 6:**
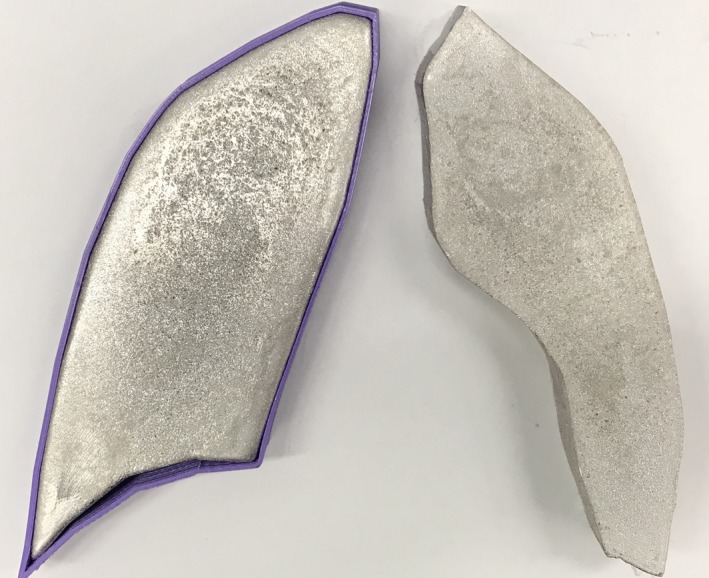
Photograph of sample lung blocks with and without the PLA form.

Our current clinical regimen for high‐dose TBI is 12 Gy delivered in 6 fractions over 3 days with greater than 6 hr between daily fractions. The dose calculated at the center of the lung has traditionally been limited to less than 8 Gy by blocking the lungs. This regimen and/or 12 Gy in 8 fractions over 4 days has been delivered at our institution for over 20 yr with negligible interstitial pneumonitis (IP) rates. Similarly, several studies have shown that IP rates become negligible regardless of dose rate as long as the total lung dose remains under 8–9 Gy.^17^, ^19^, ^20^, ^21^, ^22^ We implement lung blocking on day 2 of the regimen and thus block the lungs for 4 of 6 treatment fractions to reduce the lung dose and thus the risk of IP. Since our measured doses within the center of the lung for unblocked AP/PA treatment fields are approximately 15% higher than the rest of the body, we require a transmission factor of 0.375 to reduce this lung dose to approximately 8 Gy, assuming that blocking will be present for four of six fractions. Based on our water phantom scans in this beam at typical mid‐patient depths, including the measured 3.2% attenuation of the lung block tray, a lung block thickness of 2.5 cm is required to reduce the dose at the center of the lung to 8 Gy. Now that we are able to calculate volumetric dose data, we are able to evaluate the mean lung dose for this delivery. Typical mean doses are approximately 9 Gy for the entirety of both lungs. Figure 6 shows a photograph of the lung blocks created for the end‐to‐end testing with and without the PLA form. The forms can be printed to and blocks poured to any desired thickness in the case that we choose to use a different blocking regimen or total dose to the lungs. While the lung block tray has three sets of brackets allowing three different height settings, we chose to only use the highest setting which is 36 cm from the treatment couch. The other two sets of brackets were removed to eliminate the possibility of setting up the block tray at the wrong height. The imaging panel is used to verify the lung block positioning prior to treatment.

**Figure 7:**
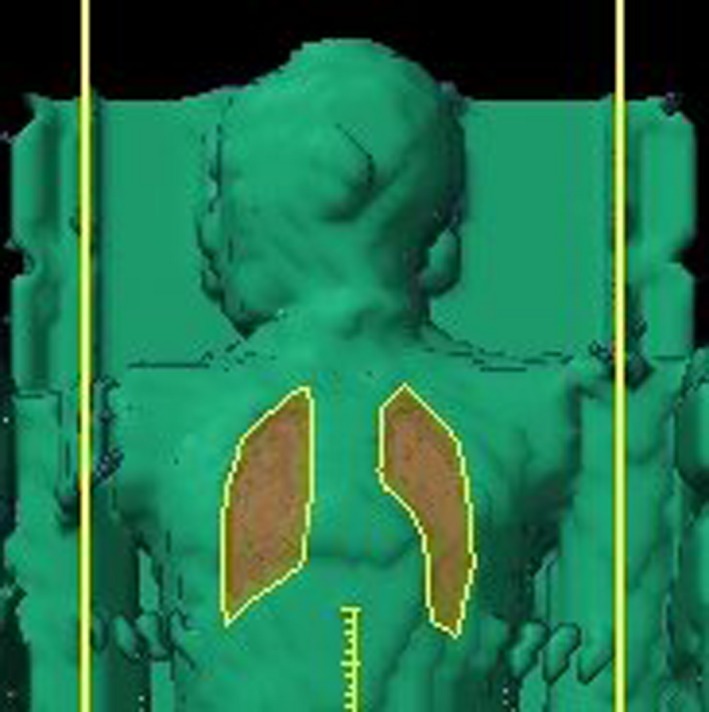
AP view of the end‐to‐end phantom with the projected lung block shapes.

**Figure 8:**
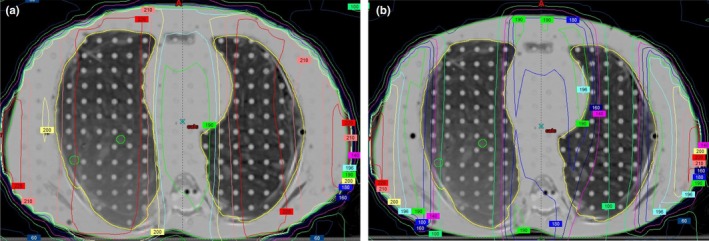
Axial MC‐calculated dose distributions (a) without and (b) with 2.5 cm thick lung blocks. The green contours within the right lung represent the “lung” and “chest wall” OSLD locations.

**Figure 9:**
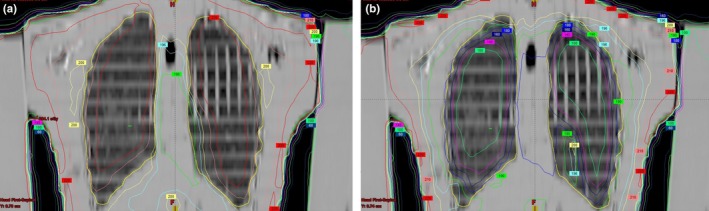
Coronal MC‐calculated dose distributions (a) without and (b) with 2.5 cm thick lung blocks. The green line in the right lung represents the “lung” OSLD location.

End‐to‐end testing was repeated with the 2.5 cm thick lung blocks described above and Table 2(b) shows calculated and measured doses at depth in the phantom for a prescribed delivered dose of 200 cGy with lung blocking in place. Measured absorbed doses agree with calculations to within 5%. Chest wall doses are not included since they are in the high‐gradient penumbral region of the lung block. As expected, the doses to the head and abdomen remained unchanged (within 1%) and the ratios of the dose in the blocked lung to the unblocked lung are 0.36 and 0.37 for the Monte Carlo calculated and measured doses, respectively. These can be compared with our transmission design criterion of 0.375. Figure 7 shows the AP view of the end‐to‐end phantom along with the projected shapes of the lung blocks contoured within the Eclipse treatment planning system. Figures 8(a) and 8(b) show axial MC‐calculated dose distributions (a) without and (b) with the 2.5 cm thick lung blocks, and Figs. 9(a) and 9(b) show the corresponding coronal dose distributions.

**Figure 10:**
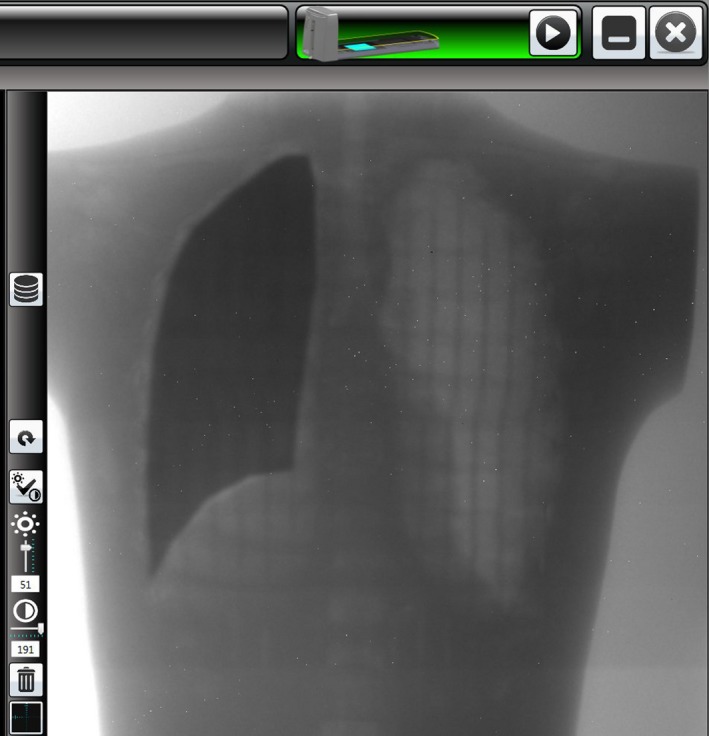
Screen capture of imaging subsystem user interface showing an AP image of the end‐to‐end phantom with the right lung blocked and left lung unblocked.

The best combination of imaging parameters for observation of the features of the contrast‐detail phantom was a gain setting of 1pF and integration time of 285 ms. However, there were not starkly observable differences between images obtained with other parameters. This combination also appeared optimal for observing lung/tissue contrast and allowing placement of the lung blocks. Figure 10 shows a screen capture of the imaging subsystem user interface and shows an AP image of the end‐to‐end phantom with the right lung blocked and left lung unblocked to show the contrast between normal tissue, lung, and lung block.

QA procedures, frequencies, and tolerances developed for this treatment unit are listed in Table 3. These satisfy our standard department procedures, and are similar to AAPM recommendations for other radiotherapy delivery devices for characteristics that these devices have in common, and satisfy regulatory requirements of the USNRC described in Title 10 of the United States Code of Federal Regulations.^23^

**Table 3:** QA procedures, frequencies (D = Daily, M = Monthly, A = Annual), and tolerances developed for the GB500 unit

Procedure	Frequency	Tolerance
Dosimetry		
Output constancy	D/M/A	3%(D), 2%(M/A)
Output calculation check	M/A	2%
External dosimetric validation	A	2%*
Source translation (“timer”/“on/off”) error	M/A	≤0.003 min
Source translation timer logs	M/A	Constancy
Timer linearity	M/A	1%
Secondary (external) timer check	M/A	1%
Off‐axis ratios	M/A	2%
Light/radiation field alignment	M/A	5 mm(M), 2 mm(A)
Calibration of *in vivo* dosimetry system	M/A	2%
Mechanical		
Laser localization	D/M/A	2 mm
Couch positioning	D/M/A	2 mm
Distance indicator	D/M/A	2 mm
Flattening filter	D/M/A	Locked in place
Head rotation	D/M/A	Locked in place
Head height	D/M/A	2 mm
Air pressure	D/M/A	≤2 psi from baseline
Couch operation	D/M/A	Functional
Light field / laser / couch alignment	D/M/A	2 mm
Imaging system	D/M/A	Functional
Image quality	M/A	Constancy
Safety		
Door interlock	D/M/A	Functional
Emergency stops	D/M/A	Functional
Beam on indicators	D/M/A	Functional
Audiovisual systems	D/M/A	Functional
Area radiation monitor	D/M/A	Functional
Survey meter	D/M/A	Functional
Battery back up power	D/M/A	≥24 V
Door operation from inside room	M/A	Functional
Emergency shutter system	M/A	Functional

*For independent ion chamber validation, or relevant satisfactory agreement for independent organization (e.g., IROC).

## DISCUSSION

Measured and calculated dosimetric results are well within the 10% uniformity in prescribed dose recommended by AAPM Report #17. Stability of the source translation process results in very reproducible translation times and timer error. In addition, the treatment field is exceptionally uniform as a function of position, yielding a dose variation of only 5% over the entire treatment field for patients as tall as 178 cm, and within 10% for patients as tall as 225 cm.

Two identified treatment field issues are the effects of the treatment couch head and the corner light field holes on the dose distribution. The treatment couch head (the housing for the couch vertical drive mechanism) increases the scattered dose to patient anatomy in close proximity. This effect was measured by reversing the end‐to‐end phantom and repeating the measurements while factoring out the off‐axis ratios without the couch present. The couch head increases the surface diode reading on the phantom head by nearly 5% for the end‐to‐end phantom setup. For very tall patients, this effect can be greater than 10% on the surface. With the phantom head touching the couch head, we measured an increase of 11.5% in the dose at d_max_ due to scatter from the couch head. For measurements at depth, the effect is only approximately 1.4%. However, this along with the difficulty modeling the longitudinal profile near the penumbral region, likely represent the majority of the difference between measured and calculated doses inside the phantom head. These are the only two of the seven comparison points which show a disagreement of greater than 2%. Backscatter off the treatment couch is also a potential source of dose calculation inaccuracy. The MC dose calculation includes the simulation couch but this may produce significantly different backscatter than the treatment couch. To evaluate this, we made diode measurements underneath the phantom and these matched the MC calculations to within 3%.

While the small light field holes result in, at most, a 2% increase in the local dose near the lateral beam penumbra, the corner holes are significantly larger and result in a dose increase of approximately 25% under these holes. As a result, users should be careful not to allow any part of a patient to approach this region. This is very unlikely given the distance of these light holes from the central axis. At a typical midplane treatment location of 10 cm up from the treatment couch surface, the corner light holes project to a distance of approximately 100 and 35 cm off axis in the longitudinal and transverse directions, respectively.

A clinically relevant design issue with the imaging system is that the superior edge of the imaging panel is currently limited to a maximum travel distance of 65.5 cm from the physical center of the treatment couch. For patients centered about the central axis of the treatment beam, this distance is often not sufficient to image the apex of the lungs. While this can be overcome by moving the patient inferiorly along the treatment couch, the patient is then closer to the edge of the usable treatment field and the patient's feet may extend off the end of the treatment couch. One other design feature that could be improved is the manual backup operation of the emergency shutter. The emergency shutter is designed to deploy automatically in the case that the source is stuck in the exposed position. However, in the event that the shutter does not deploy automatically, manual operation must be performed from the treatment unit gantry stand. Ideally, users could be given the option to manually close the emergency shutter without entering the room and coming in close proximity to the active treatment beam.

## CONCLUSIONS

A novel dedicated TBI unit has been commissioned and clinically implemented. Its mechanical, dosimetric, and imaging capabilities are suitable to provide state‐of‐the‐art TBI for patients as large as 225 cm in height and 78 cm in width. The uniformity of this treatment field is within 10% over this stated field size and within 5% over the central 178 cm and 73 cm in the longitudinal and lateral directions, respectively. All characteristics of this unit provide adequate capability to deliver TBI in accordance with tolerances recommended by AAPM Report #17.

## CONFLICTS OF INTEREST

The authors have no actual or potential conflicts of interest for the work presented here.
